# Endovascular management of RCC in one-kidney patient: a case report study

**DOI:** 10.1097/MS9.0000000000000559

**Published:** 2023-04-01

**Authors:** Amjad Ghareeb, Bairak Salameh, Malaz Halaweh, Muhamad Al-Tawil, Mohammed Ali Nahas

**Affiliations:** a Faculty of Medicine, Damascus University, Damascus, Syria; b Urology Department, Al-Mouwasat University Hospital, Damascus University, Damascus, Syria; c Chief of Vascular and Endovascular Surgery Department, Al-Assad University Hospital, Damascus University, Damascus, Syria

**Keywords:** embolization, endovascular intervention, nephrectomy, renal cell carcinoma, quality of life

## Abstract

Renal cell carcinoma is considered one of the most common and deadly cancers, which remain asymptomatic until the late stage, needing total nephrectomy when it is detected. In mono-kidney patients this will lead to hemodialysis and later a kidney transplant.

**Case presentation:**

This case represents our center’s experience and strategy in managing renal cell carcinoma in a one-kidney patient by endovascular management first followed by partial nephrectomy.

**Clinical discussion:**

The patient’s postoperative follow-up has a good quality of life with no signs of tumor recurrence or metastasis in addition to normal kidney functions tests.

**Conclusion:**

Preoperative endovascular intervention can represent a good and accepted solution for a partial nephrectomy preserving not only a normal renal function, without the need for kidney transplantation, but also a good quality of life.

## Introduction

Highlights

- Renal cell carcinoma is considered as one of the most common and deadly cancers in the world.
- Partial nephrectomy and radical nephrectomy are considered the treatment of choice for localized renal cell carcinoma.
- In one-kidney patients radical nephrectomy means the inevitable need for a hemodialysis or a kidney transplant.
- Preoperative endovascular intervention in one-kidney patient can represent a good opportunity to partial nephrectomy, which preserves a normal renal function without the need to kidney transplantation and a good quality of life.

Renal cell carcinoma (RCC) represents more than 85% of malignant renal tumors and the (5th–5% / 8th–3%) most frequently diagnosed cancer in men and women, respectively, in the United States of America. The average age of RCC diagnosis is 64 years, with a frequency ratio of men and women (1.7 : 1)^1^–^3^. It is diagnosed late because more than half of cases are asymptomatic and discovered incidentally via ultrasound or computed tomography (CT), when it is symptomatic it may present with visible hematuria, flank pain, and a palpable abdominal mass constituting the classic triad of RCC, which remains a rare appearance nowadays^4^. RCC in a solitary kidney is rather rare and presents a clinical challenge to urologists. Surgery, including partial nephrectomy (PN) and radical nephrectomy (RN), is considered the treatment of choice for localized RCC^5^. PN is now the standard surgical treatment for T1 tumors. Improved knowledge and techniques allow nephron-sparing surgery, reducing the risk of cardiovascular events, and morbi-mortality related to impaired renal function^6^,^7^. Although renal artery embolization (RAE) in the management of RCC is controversial^8^,^9^, preoperative RAE can have many benefits, which will be discussed later in this article.

This paper presents a rare case of a female patient with a history of unilateral left traumatic RN developed RCC in the other kidney managed successfully with RAE followed by PN conserving a normal patient renal function and maintaining a good quality of life. This case report has been reported in line with the Surgical CAse REport (SCARE) Criteria^10^.

## Case presentation

A 47-year-old female was admitted to the hospital due to pain in the right lumber region that started 4 months ago and was associated with weight loss and nocturnal fever. She has a surgical history for early childhood left RN due to traumatic kidney injury.

**Figure 1:**
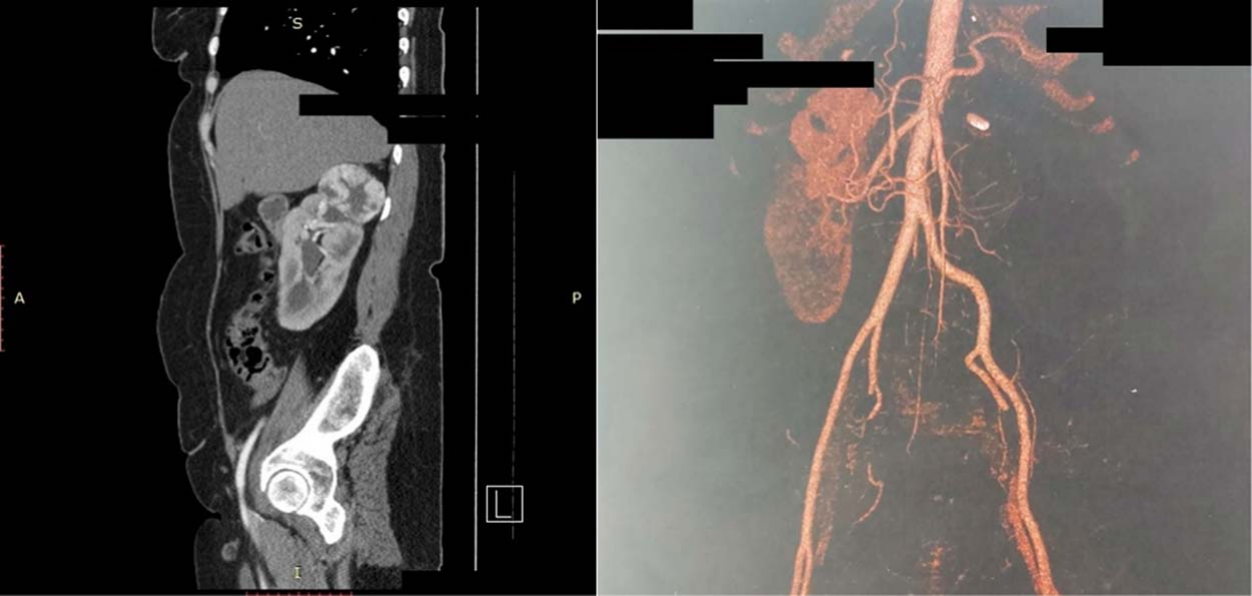
Preoperative contrast Multislice computed tomography.

The clinical examination was normal. The patient’s blood pressure was 125/85 mmHg, creatinine 0,9 mg/dl, urea 23 mg/dl. CT-scan with contrast media (Fig. 1) shows compensatory enlargement (13 cm) of the right kidney and upper renal lobe heterogeneous density mass reinforcing the contrast media in a macular shape, measuring ~6,5×4,5 cm. The left kidney was absent. The R.E.N.A.L. nephrometry score was 9× (moderate-complexity mass). A conservative strategy was adopted in order to save the renal function and avoid the need for RN by avoiding hemodialysis or renal transplantation. A presurgery embolization of the upper lobe tumor was decided.

**Figure 2:**
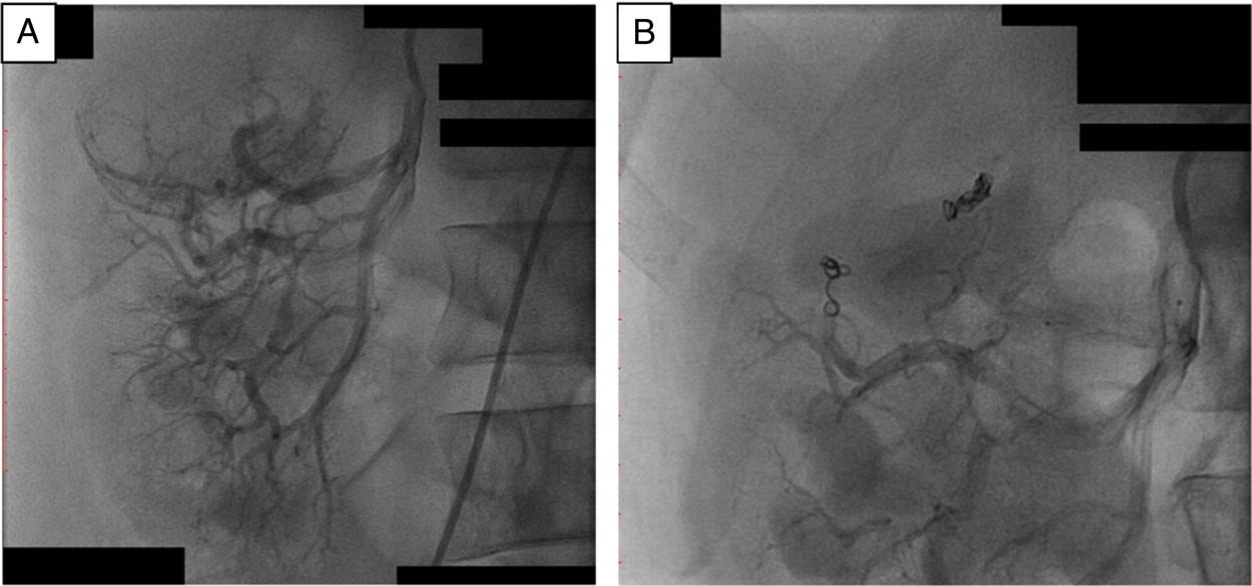
A; Pre-embolization selective Digital Subtraction Angiography, B; postembolization selective Digital Subtraction Angiography.

**Figure 3:**
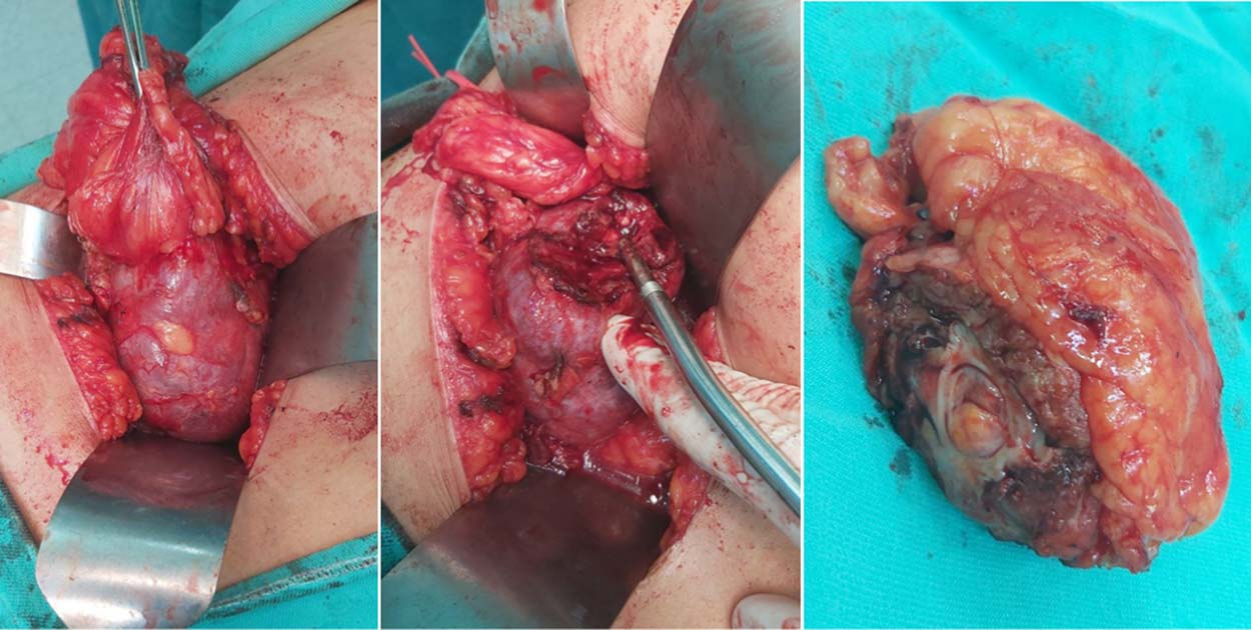
Presurgical and postsurgical excision.

Through the right common femoral artery, a 6F sheath was deployed and then using a 6F vert catheter the right renal artery was selectively catheterized. The angiographic study showed that the tumor was fed by two arteries. A 6F JR guide catheter was used and then a headway 17 Advanced Soft Microcatheter was advanced through the right catheter selectively in each one of the above two arteries, a 4 mm/12 cm MicroPlex 10 Cosmos Complex and 3 mm/6 cm MicroPlex 10 Cosmos Complex coils were deployed in the feeding arteries of the tumor getting a total devascularization of the tumor, which has been removed surgically the day after (Fig. 2). Surgery was held under general anesthesia via flank retroperitoneal incision(Fig. 3), blood loss was about 200 ml, no hilar clamping was necessary. No secondary bleeding or embolization was reported. Histo-pathology examination showed clear-cell RCC (Grade I) (pT1bNxMx); 4.5 cm limited to the excised renal upper lobe, no invasion of the renal capsule, the perirenal fat, or the excision margins. No evidence of vascular invasion.

The postoperative clinical patient status showed normal values of creatinine, urea, electrolytes, blood pressure, and urine output. The patient was dismissed after 48 h.

A 48 month follow-up confirmed normal renal function (creatinine 1.02 mg/dl and total GFR 80  ml/s compare with 89 ml/s preoperatively) and CT-scan showed no metastatic lesions.

## Discussion

RAE was described for the first time in 1969 by Halli AF and Peterson N; it was indicated in the palliative treatment of metastatic renal cancer in addition to the symptomatic treatment of hematuria^11^,^12^. Preoperative embolization avoids peri-procedural hilar clamping commonly used in PN procedures for bleeding control, in order to decrease surgical risk factors compromising renal function after PN^13^,^14^.

Actually, RN and PN are the golden standard therapy of RCC^5^,^8^,^15^ and for the first time in the literature, we are going to report this case about preoperative RAE followed by PN in a 47-year-old woman with developed RCC in one-kidney while the other one was excised.

RAE is used to reduce operative blood loss, tumor vascularity, debulk the tumor in case of nonsurgical patients, decreased operative times, and palliate symptoms such as hematuria^8^,^9^. RAE also allows surgery with zero ischemia time in order to preserve renal function^16^.

Technically, the common femoral artery is considered as the vascular access in most cases also axillary or brachial artery may be used as an alternative access. The materials used in RAE include particles, metallic coils, and sclerosants (liquids) like ethanol^17^.

Antibiotic coverage is recommended before RAE in addition to laboratory screening specially platelets (>50×103 /ul), INR (<1.5) and GFR^8^,^17^.

The appropriate time between embolization and surgery is less than 48 h, resulting in a well-tolerated RAE, reducing complications such as postembolization syndrome characterized by fever, severe flank pain, nausea, and the raising of white blood cells, this may occur 1–3 days after RAE, and can be avoided by reducing the RAE – surgery interval. Infections related to RAE are fortunately uncommon^17^,^18^.

## Conclusion

Selective RAE followed by PN can be an effective method in managing confined RCC, especially in patients with one functional kidney. This procedure is more difficult when the tumor is localized in the central parts of the kidney. We believe that the continuous progress in low-profile catheters and the embolizing materials will have a positive impact on the therapeutic strategy of these tumors resulting in more conservative approaches, which reflect less need to hemodialysis and kidney transplantation while maintaining a good quality of life.

The case has not been presented at a conference or regional meeting.

## Ethical approval

This study has been approved by the ethical committee of Damascus University.

## Patient’s consent

Written informed consent was obtained from the patient for publication of this case report and accompanying images. A copy of the written consent is available for review by the Editor-in-Chief of this journal on request.

## Sources of funding

There were no sources of funding.

## Author contribution

A.G.: data curation, resources, investigation, formal analysis, writing - review and editing contributions, software, methodology; B.S.: data curation, resources, writing - review and editing contributions, methodology; M.H.: software, methodology, resources; M.A-T.: supervision, methodology, validation; M.A.N.: conceptualization, project administration, supervision, formal analysis, validation.

## Conflicts of interest disclosure

All the authors declared that they have no conflicts of interest.

## Research registration uniqueidentifying number (UIN)

1. Name of the registry: NA.
2. Unique Identifying number or registration ID: NA.
3. Hyperlink to your specific registration (must be publicly accessible and will be checked): NA.

## Guarantor

Amjad Ghareeb is the guarantor.

## Data availability statement

All data are available from the corresponding author on reasonable request.

## Provenance and peer review

Not commissioned, externally peer reviewed.
